# Village Anthropology

**Published:** 1891-09-26

**Authors:** 


### THE DOCTOR'S ARMCHAIR.

### VILLAGE ANTHROPOLOGY.

A PHYSIOLOGICAL statesman—a statesman-like physiologist—are there any such people in this country? There ought to be. The average Briton dearly loves things in the concrete. How is it that so many of our fellow-men who enter the learned professions so rapidly divorce themselves from every-day actualities, and are content to live, move, and have their being in an immeasurable cloud of words? If it be true that on earth there is nothing great but man, are we to understand that it is the man dead, with his dead bones, and his old-world thoughts, that are alone great and interesting? The dead man, with his dead bones, and his old-world thoughts, was once a living man, and it was when he was living that he developed his bones and produced his thoughts. If he had never lived he would never have had either bones or thoughts. "A living dog is better than a dead lion," says an Eastern proverb. Nothing of the kind, answers our modern anthropologist. A dead bone, or at least a dead skeleton, is worth more than whole millions of living men and women who merely do the work and produce the bread which constitutes our daily food.

The living man we contend for, with his living body and his living soul. The metaphysician tells us that he cannot understand man without considering him in the mass as well as in the individual. If it is desired to take a just view of the human mind, then it is imperative not only to consider the mind of a single man, but of groups of men, of communities acting in concert, of races, or even of continents. What is true for the metaphysician is true for the anthropologist and the physiologist. He must not content himself with merely working in his own little laboratory. He must come out into the laboratories of nature, into families, villages, towns, and populations. The physiologist prides himself upon being a man of science; whereas in many cases he is merely a picker-up of physiological pins. It has never yet entered into his mind to take large general views. He is so small that he is not even aware there are any general views to be taken. The world is eager for physiological knowledge, otherwise it would not consent to tolerate the miserable littleness of the average physiologist.

It has been reserved for a daily newspaper, a newspaper that professes no specialty, that has no knowledge, except general knowledge, either of anthropology or physiology, to take up for England the study of man in the mass. The *Daily News* has employed an intelligent journalist, with no pretensions to physiological or medical learning, to do, thus late in the day, what doctors and physiologists, to say nothing of parsons, ought to have done a hundred and fifty years ago. The journalist has a truly scientific temper. Apparently he does his utmost to avoid extenuating anything, or setting down aught in malice. But what a story he has to tell! Villages are considered to be places where health reaches its highest level of perfection; where stalwart men and rosy children spend the evening hours playing on the green, and comely women knit in the twilight at the open cottage doors. That is the ideal picture: what is the reality? It is such as the special commissioner of the *Daily News* has described. The present writer has seen hundreds, probably thousands, of village children as pale, ricketty, and fleshless looking as the most melancholy denizens of the city slums. How can children who live in the pure air of the country be so pitiful in physique? Because the two main health conditions, of pure air and abundant food, are not fulfilled for them. The wretched little sleeping-rooms in which country children spend twelve of their twenty-four daily hours are little better than poison dens. In size an average cottage bed-room is only large enough for one person to sleep in, or for two at the most; yet the majority of such bed-rooms are made to accommodate four, five, six, or even more. The occupants of such bed-rooms simply stifle and poison one another every night. As for the food of labourers' children in villages, it is a miracle that the boys and girls live and grow up on it at all. This statement needs no argumentation. Every man with a family knows what fifteen shillings a week will do towards providing plain wholesome food for a man, his wife, and four or five children. It is not too much to say that the average labourer does not possess more than half the physical strength which he ought to possess, and which he would possess if he were sufficiently fed. The women and the children are proportionately feebler still. These facts the writer knows full well, both as a medical man, and as a lifelong student of the country poor and their ways.

Unconsciously, a little indignation animates the pen and ink as one writes on this subject. But there is no intention here of denouncing landlord, farmer, parson, or the members of any other class: unless, indeed, it be those dilettante anthropologists who have so much time to spare for the examination and discussion of fossil bones, but never give a single half hour to the consideration of the living men, women, and children of their own country. The anthropologists, with their fellow-scientists, the laboratory physiologists, require to be wakened out of their frosty and comparatively profitless hibernations by a little moral dynamite. But, as for landlords, farmers, clergymen, and labourers, they must be considered to have drifted into their present condition unconsciously, and by the almost inevitable force of historical circumstances.

But now that the facts are brought to light the evils must be remedied. The English people are one race, not two or three. They are brothers in blood. They have the same character, the same virtues, and the same faults. It is the highest interest of the nation that all its ranks and classes should stand solidly together as one people. We cannot afford to make outcasts: above all, we cannot afford that the larger half of the nation shall be outcast. The truth is coming clearly into view: it is for those who have intellectual capacity and influential position to see it, and to deal with it like statesmen and patriots. The powerful, the wealthy, and the leisured classes must take the lead. The poor cannot lead. But though they cannot lead, they can drive: and ignorant and angry people, when they take to driving, sometimes drive a great deal too fast and a great deal too far. The rich and the prosperous have an urgent call made upon them at the present crisis for statesmanship and patriotism. They will be very unlike the great Englishmen of past generations if they do not show themselves ready and equal to an occasion which is without parallel in the long and varied story of the English race.

